# Validation of a long-read 16S rRNA-gene sequencing approach for analysis of clinical samples and bacterial identification in a routine clinical laboratory

**DOI:** 10.3389/fmicb.2026.1870167

**Published:** 2026-07-30

**Authors:** Otto Van de gaer, Reinoud Cartuyvels, Timo Froyen, Petra Hilkens, Koen Magerman, Steven Martens, Britt Van Meensel, Jozef Dingemans

**Affiliations:** 1 Department of Laboratory Medicine, Jessa Hospital, Hasselt, Belgium; 2 Department of Immunology and Infection, Hasselt University, Hasselt, Belgium

**Keywords:** 16S sequencing, diagnostics, Nanopore, routine, validation

## Abstract

**Background:**

Currently, microbiological diagnosis relies heavily on classical culture-based techniques, which have notable limitations. These methods often struggle with detecting non-culturable or slow-growing microorganisms and can yield negative results in patients who have already received antimicrobial treatment before sample collection. 16S rRNA gene sequencing offers an alternative to culture-based methods, enabling bacterial identification without the need for cultivation. This study presents the validation and clinical implementation of a 16S rRNA-gene Nanopore-based sequencing workflow in a routine hospital.

**Methods:**

Estimated limit of detection and linearity were investigated using spiking experiments with a dilution series of ATCC strains and commercial quality control material. Accuracy and including taxonomic resolution was evaluated for 37 strains obtained from external quality control programs and 30 clinical from a clinical collection. Utility was investigated by comparing 16S rRNA gene Nanopore-based sequencing with culture results and other clinical findings for 54 prospectively collected clinical samples.

**Results:**

An estimated limit of detection of 260 and 747 CFU/ml was found for respectively *Staphylococcus aureus* and *Pseudomonas aeruginosa* in synovial fluid. R^2^ analysis ranged from 0.9778 to 0.9986 depending on sample matrix. Correct genus level identification of strains was >98% and correct species level identification was >85%. 25/26 clinical culture-positive samples tested positive with 16S sequencing. Additional clinically relevant pathogens were detected in 5/28 culture-negative and 7/26 culture-positive samples.

**Discussion:**

This study demonstrates that the implementation of 16S rRNA gene sequencing is feasible in a routine clinical laboratory setting and provides additional utility for the identification of bacterial isolates and direct detection on clinical samples, increasing recovery of non-culturable isolates.

## Introduction

Timely and accurate identification of bacterial pathogens is essential in clinical microbiology, as delay in diagnosis is associated with increased morbidity, mortality, and inappropriate antimicrobial use (Martínez et al., 2020). In routine diagnostic laboratories, bacterial identification is typically performed through culture of clinical specimens followed by biochemical characterization or matrix-assisted laser desorption ionization–time of flight mass spectrometry (MALDI-TOF-MS). Although these approaches are robust and widely implemented, their reliance on viable organisms imposes inherent limitations. Culture-based methods are time consuming and may lack sensitivity, particularly for slow or non-culturable organisms or in patients who have received empiric antimicrobial therapy prior to sample collection (Church et al., 2020; Sigakis et al., 2019).

Sequence-based techniques offer an important alternative by enabling detection and identification of bacterial DNA of isolates as well as clinical samples without the need for prior cultivation (Botan et al., 2024). While traditional partial sequencing approaches such as Sanger sequencing have limited resolution for closely related species, long-read platforms such as Nanopore-based technology enable full-length (∼1,500 bp) sequencing of the 16S rRNA gene, improving taxonomic accuracy (Campodónico et al., 2025).

In the past years, several studies have demonstrated the utility of long-read 16S rRNA gene sequencing in resolving culture-negative infections, supporting clinical decision-making in complex cases (Lao et al., 2024; Vanhee et al., 2024) and identifying bacterial isolates (Campodónico et al., 2025; Lao et al., 2022). Historically, implementation has been largely restricted to specialized reference laboratories due to cost, technical complexity, and bioinformatics requirements. However, advances in long-read next-generation sequencing technologies have improved accessibility and reduced costs, facilitating broader adoption in routine diagnostic settings (Brunet et al., 2025).

In this study, a full-length Nanopore-based 16S sequencing workflow was validated and implemented in a routine clinical laboratory with the goal of improving recovery of pathogens. Estimated limit of detection, linearity and reliable identification of a selection of pathogens were evaluated as primary endpoints. Clinical utility was assessed as a secondary outcome using a set of representative clinical samples.

## Materials and methods

### Study design and setting

This validation study was performed in Jessa Hospital, a 981-bed regional teaching hospital in Belgium. The study period spanned from October 2025 to February 2026. Microbiology work-up and sample preparation was performed at the clinical microbiology laboratory. DNA amplification, sequencing and bioinformatic analysis took place in the in-house molecular laboratory.

### Linearity and estimated limit of detection

Linearity was determined by separately spiking *Staphylococcus aureus* (ATCC 43300) and *Pseudomonas aeruginosa* (ATCC 27853) with the ZymoBIOMICS Spike-in Control II (Low Microbial Load) in negative cerebrospinal fluid (CSF) and synovial fluid matrix. The spike in control consisted of a mix of *Truepera radiovictrix*, *Imtechella halotolerans* and *Allobacillus halotolerans* in well-defined quantities. The theoretical load of *S. aureus* and *P. aeruginosa* was calculated for a 5-step dilution series starting from a 0.5 McFarland suspension and compared to the estimated load as measured by sequencing after correcting for 16S duplicates in a genome. Analysis was performed once for each combination of a specific bacteria, dilution and matrix.

The estimated limit of detection (CFU/ml) was calculated by comparing *S. aureus* and *P. aeruginosa* reads to the lowest spike-in control (*Allobacillus halotolerans*) after normalizing the total read count to 2500 to reflect feasible routine sequencing practice (Supplementary Figure 1 and Supplementary Table 1). A cut-off of 25 reads corresponding to at least 1% of the total amount of bacterial reads was chosen to enable consistent quantification. The choice of this cut-off was based on previous literature which showed that a 1% abundance threshold was able to significantly reduce background noise from contaminants, in particular for low microbial biomass samples (Bouchiat et al., 2022; Karstens et al., 2019).

### Sample collection - documented strains

Thirty-seven well defined bacterial species obtained from external quality control programs and twenty-nine bacterial species obtained from clinical samples were cultivated on solid agar media following standard protocol after storage at −80 °C. Identification was verified by MALDI-TOF MS (MALDI Biotyper^®^ Sirius, Bruker) analysis (Table 1). For molecular processing 250 μl of a 0.5 McFarland suspension was added to DNA/RNA Shield Lysis Tubes (Microbe, Zymo Research) and sent for DNA extraction.

**TABLE 1:** Species identification of 37 reference strains and 29 clinical strains by 16S Nanopore-based gene sequencing and BugSeq analysis.

ID	Origin	Species	BugSeq result
1	WIV / EQC	*Abiotrophia defectiva*	*Abiotrophia defectiva*
2	WIV / EQC	*Achromobacter xylosoxidans*	*Achromobacter xylosoxidans*
3	QCMD / EQC	*Acinetobacter baumannii*	*Acinetobacter baumannii*
4	WIV / EQC	*Actinotignum schaalii*	*Actinotignum timonense*
5	Clinical / MALDI	*Aerococcus sanguinicola*	*Aerococcus sanguinicola*
6	Clinical / MALDI	*Aeromonas caviae* complex	*Aeromonas caviae*
7	Clinical / MALDI	*Alcaligenes faecalis*	*Alcaligenes faecalis*
8	WIV / EQC	*Bacillus cereus*	*Bacillus cereus group*
9	UKNEQAS / EQC	*Bacteroides fragilis*	*Bacteroides fragilis*
10	ATCC	*Bacteroides thetaiotaomicron*	*Bacteroides thetaiotaomicron*
11	Clinical / MALDI	*Bifidobacterium scardovii*	*Bifidobacterium scardovii*
12	WIV / EQC	*Burkholderia multivorans*	*Burkholderia multivorans*
13	Clinical / MALDI	*Campylobacter jejuni*	*Campylobacter jejuni*
14	Clinical / MALDI	*Capnocytophaga sputigena*	*Capnocytophaga sputigena*
15	ATCC	*Citrobacter freundii*	*Citrobacter freundii*
16	Clinical / MALDI	*Citrobacter koseri*	*Citrobacter koseri*
17	ATCC	*Clostridiodes difficile*	*Clostridioides difficile*
18	Clinical / MALDI	*Comamonas kerstersii*	*Comamonas kerstersii*
19	WIV / EQC	*Corynebacterium diphtheriae*	*Corynebacterium belfantii*
20	Clinical / MALDI	*Corynebacterium striatum*	*Corynebacterium striatum*
21	WIV / EQC	*Corynebacterium ulcerans*	*Corynebacterium ulcerans*
22	WIV / EQC	*Dermabacter hominis*	*Dermabacter jinjuensis*
23	UKNEQAS / EQC	*Eikenella corrodens*	*Eikenella halliae*
24	Clinical / MALDI	*Enterobacter asburiae*	*Enterobacter kobei*
25	WIV / EQC	*Enterobacter cloacae*	*Enterobacter hormaechei*
26	QCMD / EQC	*Enterococcus faecium*	*Enterococcus faecium*
27	QCMD / EQC	*Escherichia coli*	*Escherichia coli*
28	ATCC	*Haemophilus influenzae*	*Haemophilus influenzae*
29	Clinical / MALDI	*Hafnia alvei*	*Hafnia alvei*
30	WIV / EQC	*Hathewaya histolytica*	*Hathewaya histolytica*
31	WIV / EQC	*Kingella kingae*	*Kingella kingae*
32	WIV / EQC	*Klebsiella aerogenes*	*Klebsiella aerogenes*
33	QCMD / EQC	*Klebsiella pneumoniae*	*Klebsiella pneumoniae*
34	Clinical / MALDI	*Lactobacillus jensenii*	*Lactobacillus jensenii*
35	Clinical / MALDI	*Lactococcus garvieae*	*Lactococcus garvieae*
36	Clinical / MALDI	*Leptotrichia trevisanii*	*Leptotrichia trevisanii*
37	Clinical / MALDI	*Lysinibacillus sphaericus*	*Lysinibacillus fusiformis*
38	Clinical / NRC	*M. chelonae*-absc complex	*Mycobacteroides chelonae*
39	Clinical / MALDI	*Micrococcus luteus*	*Micrococcus luteus*
40	Clinical / MALDI	*Morganella morganii*	*Morganella morganii*
41	Clinical / MALDI	*Morganella morganii*	*Morganella morganii*
42	WIV / EQC	*Mycolicibacterium fortuitum*	*Mycolicibacterium fortuitum*
43	ATCC	*Neisseria gonnorhoeae*	*Neisseria gonnorhoeae*
44	WIV / EQC	*Nocardia cyriacigeorgica*	*Nocardia cyriacigeorgica*
45	WIV / EQC	*Brucella anthropi*	*Brucella anthropi*
46	Clinical / MALDI	*Proteus mirabilis*	*Proteus mirabilis*
47	Clinical / MALDI	*Proteus vulgaris*	*Proteus vulgaris*
48	Clinical / MALDI	*Providencia rettgeri*	*Escherichia coli*
49	Clinical / MALDI	*Providencia rettgeri*	*Providencia rettgeri*
50	Clinical / MALDI	*Pseudomonas aeruginosa*	*Pseudomonas aeruginosa*
51	Clinical / MALDI	*Pseudomonas aeruginosa*	*Pseudomonas aeruginosa*
52	UKNEQAS / EQC	*Pseudomonas monteilii*	*Pseudomonas monteilii*
53	QCMD / EQC	*Serratia marcescens*	*Serratia marcescens*
54	Clinical / MALDI	*Serratia marcescens*	*Serratia marcescens*
55	Clinical / MALDI	*Serratia odorifera*	*Serratia odorifera*
56	QCMD / EQC	*Staphylococcus aureus*	*Staphylococcus aureus*
57	Clinical / MALDI	*Staphylococcus borealis*	*Staphylococcus haemolyticus*
58	QCMD / EQC	*Staphylococcus epidermidis*	*Staphylococcus epidermidis*
59	WIV / EQC	*Staphylococcus lugdunensis*	*Staphylococcus lugdunensi*
60	ATCC	*Staphylococcus saprophyticus*	*Staphylococcus saprophyticus*
61	ATCC	*Streptococcus agalactiae*	*Streptococcus agalactiae*
62	WIV / EQC	*Streptococcus equi*	*Streptococcus equi*
63	Clinical / MALDI	*Streptococcus pyogenes*	*Streptococcus pyogenes*
64	WIV / EQC	*Streptococcus salivarius*	*Streptococcus salivarius*
65	Clinical / MALDI	*Sutterella wadsworthensis*	*Sutterella wadsworthensis*
66	Clinical / MALDI	*Winkia neuii*	*Winkia neuii*
67	Clinical / NRC	*Yersinia enterococolitica*	*Yersinia enterocolitica*

66 bacterial species were cultivated and species was confirmed by MALDI-TOF-MS. Discordances are underlined.

### Sample collection - clinical samples

Clinical samples (*n* = 54) from 53 unique patients were processed either after or in parallel with routine microbiology diagnostics. Both culture-negative (*n* = 28) and culture-positive (*n* = 26) samples were included. Samples were stored at 4 °C and DNA extraction occurred up to 14 days after sample collection (mode 3 days). When necessary, tissue samples were homogenized using a gentleMACS dissociator (Miltenyi Biotec). For molecular processing 250 μl of the suspended clinical sample and 20 μl of the ZymoBIOMICS Spike- Control II (Low Microbial Load) was added to DNA/RNA Shield Lysis Tubes (Microbe, Zymo Research).

Included clinical samples consisted mostly of bone and joint tissue samples, collected from patients with suspected prosthetic joint infection (*n* = 30), along with biopsies and aspirates from various “sterile” anatomical sites (Tables 2, 3).

**TABLE 2:** Sample type, culture results and 16S Nanopore-based sequencing results of 26 culture positive clinical samples.

ID	Type	Conventional culture & MALDI-TOF	16S Nanopore sequencing & BugSeq
A	Biopsy (ear)	*S. epidermidis* *S. aureus* *M. chelonae*	*S. epidermidis* *S. aureus* *M. chelonae*
B	Sputum	*M. holsaticum*	*M. holsaticum*
C	Brain abscess	*S. anginosus* group	*S. intermedius* *F. animalis* *C. gracilis* *S. odontolytica* *F. vincentii*
D	CSF	*S. anginosus* group	*S. intermedius* *F. animalis* *C. gracilis* *F. vincentii*
E	Biopsy (Tibia)	*E. faecalis* *E. cloacae* complex *Acinetobacter* spp.	*E. faecalis* *E. cloacae* complex *Acinetobacter pittii*
F	CSF	*S. aureus*	*S. aureus* complex
G	Biopsy (Sternum)	*S. aureus*	*S. aureus*
H	CSF	*S. pneumoniae*	*S. pneumoniae*
I	Synovial fluid	*S. epidermidis*	*S. epidermidis*
J	Biopsy	*C. acnes*	*C. acnes*
K	Aortic valve	*A. urinae*	*A. urinae complex*
L	Aortic valve	*S. aureus*	*S. aureus*
M	Empyema	*S. anginosus* group	*S. intermedius* *F. vincentii* *Parvimonas* spp. *P. massilienssis*
N	Mitral valve	*S. aureus*	*S. aureus*
O	Biopsy (PJI hip)	*E. cloacae* complex *S. haemolyticus* *S. epidermidis* *P. aeruginosa* *E. faecalis*	*E. cloacae* complex *S. haemolyticus* *S. epidermidis* *P. aeruginosa* *E. faecalis* *C. marquesiae* *K. michiganensis* *D. jinjuensis*
P	Biopsy (PJI humerus)	*C. acnes*	*C. acnes*
Q	Biopsy (calcaneus)	*S. agalactiae* *E. casseliflavus*	*S. agalactiae* *E. casseliflavus* >10 anaerobic bacteria
R	Biopsy (PJI humerus)	*S. aureus* *S. epidermidis*	*S. aureus* *S. epidermidis* *Corynebacterium* spp. *Brevibacterium* spp. *F. magna*
S	Biopsy (PJI shoulder)	*S. epidermidis*	*S. hominis*
T	Biopsy (PJI knee)	*S. aureus*	*S. aureus*
U	Biopsy (PJI knee)	*E. cloacae* complex	*E. cloacae* complex
V	Biopsy (PJI hip)	*S. epidermidis*	*S. epidermidis*
W	Biopsy (PJI hip)	*S. aureus* *S. epidermidis*	*S. epidermidis*
X	Biopsy (PJI hip)	*S. epidermidis*	Negative
Y	Biopsy (calcaneus)	*S. simulans* *P. olsenii*	*S. simulans* *P. olsenii* *F. magna* *S. pettenkoferi* *S. simulans* *>10 anaerobic bacteria*
Z	Biopsy (PJI synovium)	*S. agalactiae*	*S. agalactiae*

Discordances and additional findings are underlined.

**TABLE 3:** Sample type, 16S Nanopore-based sequencing results and additional diagnostic modalities of 6 culture-negative clinical samples with positive 16S analysis.

ID	Type	16S Nanopore sequencing & BugSeq	Additional diagnostic modality
AA	Biopsy (Pericardial)	*Neisseria meningitidis*	Monoplex PCR positive
AB	Mitral valve	*Streptococcus dysgalactiae*	Positive blood culture
AC	Pacemaker lead	*Streptococcus dysgalactiae*	Positive blood culture
AD	Biopsy (Brain abscess)	*Aggregatibacter actinomycetemcomitans*	/
AE	Biopsy (PJI)	*Staphylococcus epidermidis*	3 other biopsies positive
AF	Biopsy (PJI – fracture)	*Massilia timonae*	/

### Extraction and 16S PCR

Nucleic acids were extracted after 40 min of bead beating on a Vortex Genie 2 (Scientific Industries, Inc.) at maximum speed using the ZymoBIOMICS DNA Miniprep Kit (Zymo Research), with 400 μl input and 50 μl elution. PCR amplification and library preparation were performed using the Microbial Amplicon Barcoding Kit 24 V14 (SQK-MAB114.24; Oxford Nanopore Technologies - ONT) under modified conditions, including Q5 Hot Start High-Fidelity 2X Master Mix (New England Biolabs) and 30 PCR cycles. Both negative extraction controls and no template controls were used.

### Evaluation of bias in the analytical workflow

To assess potential bias in the analytical workflow (from DNA extraction to sequencing analysis), 75 μl of the ZymoBIOMICS Microbial Community Standard (Zymo Research) was added to a DNA/RNA Shield Lysis tube (Microbe, Zymo Research) according to the manufacturer’s protocol and processed as described above. Intra-run and inter-run precision was assessed by means of processing (DNA extraction to data-analysis) the ZymoBIOMICS Microbial Community Standard II (Log Distribution) twice in the same run and once in a separate run.

### Nanopore-based sequencing and data analysis

Sequencing was carried out on a MinION R10.4.1 flow cell as per manufacturer instruction with Super Accuracy Basecalling using MinKNOW v25.09.16 (ONT). Data was analyzed using the BugSeq data analysis software (Version 6.1) and the BugRef curated database (Fan et al., 2021). Quality-score and length filtering were automatically performed according to a published protocol (Chen et al., 2018). Bacterial genera or species that were identified in simultaneously processed negative controls were removed from the sequencing results unless they were >10-fold more abundant in clinical specimens as previously utilized in another clinical metagenomics study (Lan et al., 2025). Sequencing results of clinical samples were discussed in a team of medical microbiologists and molecular biology experts taking into account conventional microbiological work-up and clinical context.

### Acceptance criteria

Acceptance criteria for implementation of the Nanopore-based 16S rRNA sequencing workflow were defined for both documented strains and clinical samples. Analytical performance was considered acceptable when at least 95% of documented strains were correctly identified at the genus level and at least 80% at the species level. Clinical performance was evaluated in comparison with a composite of conventional culture results, clinical context and other diagnostic assays, with a minimum concordance of 90% required to demonstrate acceptable diagnostic agreement.

## Results

### Linearity and limit of detection

**FIGURE 1:**
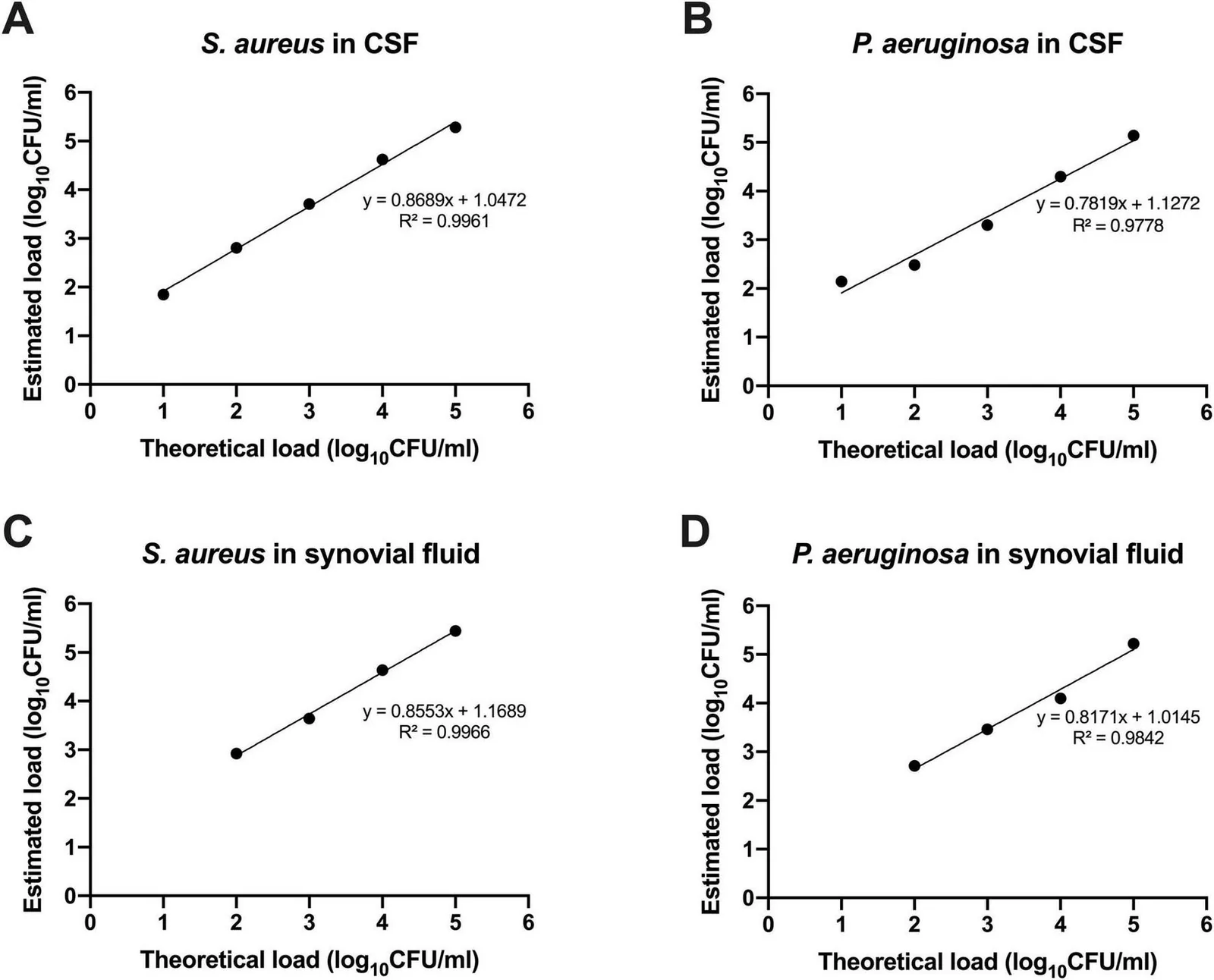
Linear relationship between the theoretical load and the estimated load for *S. aureus* in CSF **(A)**, *P. aeruginosa* in CSF **(B)**, *S. aureus* in synovial fluid **(C)** and *P. aeruginosa* in synovial fluid **(D)**. The total number of reads (per sample) was normalized to 2500 to allow standardization between experiments.

Comparing the estimated load to the theoretical load for *S. aureus* and *P. aeruginosa* in both CSF and synovial fluid, a significant linear relationship was observed. (R^2^ range: 0.9778–0.9986, Figure 1).

The limit of detection was 260 and 268 CFU/ml for *S. aureus* and 747 and 1025 CFU/ml for *P. aeruginosa* in synovial fluid and CSF, respectively.

### Evaluation of bias in analytical workflow

Potential bias in the analytical workflow (from DNA extraction to sequencing analysis) was assessed using the ZymoBIOMICS Microbial Community Standard (Zymo Research). Minimal differences (<5%) were observed between the theoretical and observed abundance for the eight bacterial species (including three Gram-negative and five Gram-positive species) present in this mock community (Supplementary Table 2). Intra-run and inter-run precision was assessed by means of the ZymoBIOMICS Microbial Community Standard II (Log Distribution) (Supplementary Table 3). All 5 most abundant species were detected and the variation coefficient ranged between 3.1% for the most abundant to 21.7% for the least abundant species.

### Identification of documented bacterial strains

Sample origin, species and BugSeq result are summarized in Table 1. Overall, BugSeq analysis yielded correct genus-level identification for 66/67 isolates and correct species-level identification for 58/67 isolates. Five discrepancies were observed for reference strains and 4 for clinical strains. A clinical *Providencia rettgeri* strain was misidentified as *Escherichia coli*. Additionally, incorrect species-level assignments were noted for both an in-house and quality control *Enterobacter cloacae* complex. Accurate species-level identification was achieved for all other Enterobacterales isolates, including another *Providencia rettgeri*, *Serratia* spp., *Klebsiella* spp., *Yersinia* spp., *Citrobacter* spp., *Morganella* spp., *Hafnia* spp., and *Escherichia* spp.

Additional species-level discordances were observed for 2 clinical strains (Lysinobacillus sphaericus and Staphylococcus borealis) and 4 quality control strains (Dermabacter hominis, Corynebacterium diphtheriae, Eikenella corrodens and Actinotignum schaalii).

### Clinical samples

A detailed overview of sample types and corresponding results of culture and BugSeq analysis of 16S Nanopore-based sequencing are provided in Tables 2, 3 for culture-positive and culture-negative samples, respectively.

### Culture positive samples

Among the 26 culture-positive samples, concordant results between conventional culture and 16S sequencing were observed in 23 cases. Notable discrepancies were seen for 3 samples, all tissue biopsies for suspected prosthetic joint infection: In sample X, a *Staphylococcus epidermidis* which was detected by culture on enrichment media was not detected by 16S sequencing. In sample S, discordance at the species level within the genus *Staphylococcus* was observed. For sample W, *Staphylococcus aureus* detected by culture across multiple media was not identified by 16S sequencing. Additionally, in 7/26 culture-positive samples, 16S sequencing identified additional bacterial taxa not recovered by conventional culture. These organisms predominantly consisted of polymicrobial anaerobic flora (Table 2).

### Culture negative samples

For the 28 culture-negative samples, BugSeq analysis of 16S Nanopore-based sequencing resulted in the detection of 6 bacteria. *Neisseria meningitidis* was recovered from pericardial tissue (sample AA, *Streptococcus dysgalactiae* from a mitral valve (sample AB) and pacemaker lead (sample AC), *Aggregatibacter actinomycetemcomitans* from a cerebral abscess (sample AD), *Staphylococcus epidermidis* from a tissue biopsy (suspected PJI – sample AE) and *Massilia timonae* from a tissue biopsy (suspected PJI – sample AF) (Table 3).

## Discussion

Despite ongoing advances in clinical microbiology, routine diagnostic workflows remain largely dependent on culture-based techniques, which can be inherently limited in sensitivity and speed, particularly for fastidious or non-culturable organisms and in the context of prior antimicrobial exposure. Sequencing of the 16S rRNA gene represents a promising culture-independent approach, and recent developments in Nanopore-based technology offer the additional advantages of rapid turnaround and flexible implementation in diagnostic laboratories. In this study, the validation and implementation of a Nanopore-based 16S rRNA gene sequencing approach was described. The primary objectives were to assess its analytical robustness and practical feasibility across relevant sample types, and the secondary objective to determine its clinical utility as an adjunct to conventional microbiological diagnostics in a real-world laboratory environment.

This study evaluated the estimated limit of detection and assay linearity, as these are critical in ensuring analytical performance and clinical reliability. The estimated limit of detection reported in this study is comparable to values reported in previous studies (Lao et al., 2024), which is higher than singleplex real-time PCR assays (typically 10–100 CFU/ml) (Papaparaskevas et al., 2013; Marimuthu et al., 2024), comparable to broad-range 16S PCR with Sanger sequencing (in case of single species infections) (Schuurman et al., 2004) and real-time multiplex PCR (100–1000 CFU/ml) (Leber et al., 2016) but lower than shotgun metagenomics approaches (≥10^3^ CFU/ml) (Barrett et al., 2020). Comparing Nanopore-based 16S rRNA gene sequencing to culture-based methods in terms of sensitivity is difficult as this is highly context-dependent. For instance, although there is no increased sensitivity for the detection of less fastidious bacteria in PJI infections, there seems to be a significant advantage in clinical contexts where patients have received prior antibiotic therapy or the presence of polymicrobial and/or anaerobic pathogens is suspected (Lewinski et al., 2023). Results should be interpreted with caution due to extrapolation of theoretical load from McFarland suspension, potentially less accurate compared to microscopic or plate counting protocols. Additionally, spiking experiments with common bacteria in CSF and synovial fluid might not accurately predict limit of detection of fastidious or difficult to lyse bacteria as well as for other sample types, limiting external validity.

Especially for the interpretation of low-abundant signals, the use of a spike-in control provides a valuable internal reference to account for variability introduced during DNA extraction, amplification and sequencing. Normalization of read counts facilitates clinical interpretation by reducing the impact of technical variation such as time spent sequencing and number of samples batched together. Monitoring of spike-in recovery across runs enables detection of deviations in assay performance as part of ongoing quality control (Tourlousse et al., 2017).

The observed variation coefficients in our study are in line with those of previously published studies and show a similar inverse relationship between the variation coefficient and the relative abundance of a particular bacterial species (Celis et al., 2022; Nikodemova et al., 2023).

This study demonstrated high genus (66/67; 98.5%) and species (58/67;86.6%) level identification of a broad collection of bacterial strains consisting of 37 strains obtained from external quality programs and 30 clinical strains, satisfying the defined acceptance criteria. A recent study evaluating Nanopore-based 16S gene sequencing for bacterial isolates that could not be identified by MALDI-TOF-MS reported similar results for a more challenging panel of strains (Campodónico et al., 2025). Another study reported slightly lower species-level identification (38/69, 55%) for a similar collection of strains. Improved identification in this study could possibly be attributed to the use of novel primers, BugSeq data analysis as opposed to EPI2ME analysis or a combination thereof (Vanhee et al., 2024). Novel Nanopore sequencing primers used for 16S PCR cover a broad range of bacterial pathogens, which we confirmed by successfully amplifying 67 cultivated bacteria. Additionally, full-length 16S sequencing increases confidence in taxonomic assignments by enabling a more comprehensive alignment and comparison to reference databases (Campodónico et al., 2025). These benefits align with recommendations outlined in the Clinical and Laboratory Standards Institute M18 guideline which emphasize the importance of sequence quality, length and percent identity thresholds for reliable taxonomic classification (Clinical and Laboratory Standards Institute [CLSI], 2018).

No clear explanation was found for the single genus-level misidentification of *Providencia rettgeri* strain. While MALDI-TOF-MS spectra showed high scores (2.41), a molecular reference identification was not performed. Successful discrimination of *Providencia* spp. and *Escherichia* spp. was expected based on primer analysis and *in silico* comparison of 16S sequences. Due to the isolate being unavailable for repeat analysis, a second *Providencia rettgeri* was included and yielded correct identification. Earlier studies have described difficulties in assigning genus level identifications for select Enterobacterales, citing recent taxonomic restructuration as a possible cause (Vanhee et al., 2024). However, due to correct genus level assignment for all other included Enterobacterales (including another *Providencia* spp.) a more isolated cause was suspected. A pre-analytical laboratory based error, where for example a different colony was sent for molecular analysis compared to MALDI-TOF-MS analysis, cannot be excluded.

Other discordances are more likely explained by (post-) analytical limitations, such as database limitations and 16S sequence similarity. Species-level misidentification of *Enterobacter cloacae* complex strains in both a clinical and reference strain possibly reflects the well-documented challenges associated with species-level discrimination within the Enterobacterales when relying on MALDI-TOF-MS and 16S rRNA gene sequences (Resendiz-Nava et al., 2021). Two additional species-level misidentifications were seen for the clinical strains *Staphylococcus borealis* and *Lysinobacillus sphaericus*. While no molecular reference identification was available for these strains, the 16S identity between both *Staphylococcus borealis* and *Staphylococcus haemolyticus* and *Lysinobacillus sphaericus* and *Lysinobacillus fusiformis* is reported to be high (>99.8%), reflecting difficulty to discriminate closely related species based solely on 16S rRNA analysis (Król et al., 2023; Meng et al., 2023). These difficulties were potentially also observed in 4 reference strains with species-level misidentifications. Specifically, *Eikenella corrodens* exhibited 98.8% 16S rRNA gene sequence identity with *Eikenella halliae*; *Actinotignum schaalii* showed 98.5% identity with *Actinotignum timonense*; *Dermabacter hominis* demonstrated 98.5% identity with *Dermabacter jinjuensis* and *Corynebacterium diphtheriae* displayed 99.0% identity with *Corynebacterium belfantii* (Bernard et al., 2020; Brahimi et al., 2017; Park et al., 2016; Schlez et al., 2021). Considering these findings, 16S rRNA gene variability and the CLSI MM18 guideline were systematically consulted prior to adopting species-level identification in routine practice (Clinical and Laboratory Standards Institute [CLSI], 2018).

The clinical utility of 16S Nanopore-based sequencing was evaluated on 26 culture-positive and 28 culture negative samples. A diagnostic summary overview is provided in Table 4. In sample X, *Staphylococcus epidermidis* was cultured, but could not be detected by 16S Nanopore-based sequencing. However, growth was restricted to enrichment media and could not be confirmed in additional biopsies from the same clinical episode, suggesting (pre-)analytical lab-based contamination as a possible explanation. In sample W, *Staphylococcus aureus* and *Staphylococcus epidermidis* were cultured while 16S Nanopore-based sequencing only detected *Staphylococcus epidermidis*. This result warrants further consideration, as *Staphylococcus aureus* was recovered from enrichment media (blood cultures) of 3 perioperative tissue biopsies and was clinically considered the causative pathogen, whereas 16S Nanopore sequencing identified only *Staphylococcus epidermidis*. Several factors could account for this discrepancy. First, in the setting of polymicrobial infection, differences in bacterial load may lead to preferential amplification and sequencing of the more abundant organism, potentially masking the presence of co-infecting species. Additionally, variability in DNA extraction efficiency, stochastic effects during PCR amplification, or primer-related amplification bias has been described to reduce the prevalence of taxa in sequencing libraries (Rauer et al., 2025). We have performed experiments using the ZymoBIOMICS Microbial Community Standard (Zymo Research) to safeguard unbiased extraction and sequencing to the extent possible (Supplementary Table 2). Additionally, sample heterogeneity between aliquots used for culture and molecular testing may have resulted in unequal pathogen distribution, particularly in samples with a low bacterial burden. For this sample, growth of *S. epidermidis* was reported after direct inoculation on solid media, while *S. aureus* was only recovered from enrichment media, possible reflecting a difference in relative abundance.

**TABLE 4:** Diagnostic summary of 16S Nanopore sequencing results compared with conventional culture.

Category	Number of samples	Findings
Culture-positive detected by 16S sequencing	24/26 – (92%)	Concordant detection of the cultured pathogen by 16S sequencing
Culture positive additional findings 16S	7/26 – (27%)	Additional recovery of anaerobic pathogens. (Brain abscess - sample C; CSF - sample D; Empyema - sample M; Biopsy – sample O, Q, R & Y)
Culture-positive missed by 16S sequencing	2/26 – (8%)	Sample W: culture-confirmed *Staphylococcus aureus* from multiple perioperative biopsies not detected by 16S sequencing; considered a true false-negative result. Sample X: *Staphylococcus epidermidis* cultured only after enrichment and not detected by 16S sequencing; considered likely contamination.
Culture-negative but clinically supported 16S-positive findings	5/28 – (18%)	*Neisseria meningitidis* (pericardial tissue, sample AA), *Streptococcus dysgalactiae* (mitral valve, sample AB; pacemaker lead, sample AC), and *Aggregatibacter actinomycetemcomitans* (cerebral abscess, sample AD); *Staphylococcus epidermidis* (PJI, sample AE) all considered clinically plausible pathogens.
Culture-negative 16S findings of uncertain clinical significance	1/28 – (4%)	*Massilia timonae* (sample AF) detected in tissue biopsies from suspected prosthetic joint infection; clinical relevance could not be established.

Additional recovery of anaerobic polymicrobial flora was seen in 7 samples. A cerebrospinal fluid, and cerebral abscess aspirate of the same patient, an empyema and 4 tissue biopsies. Anaerobic pathogens were considered relevant based on clinical evaluation and the presence of aerobic pathogens known to be associated with anaerobic polymicrobial infections such as *Streptococcus anginosus* (Pilarczyk-Zurek et al., 2025). Limited recovery of anaerobic organisms is a well-recognized limitation of conventional culture methods (Rachita et al., 2026).

For 5/6 culture-negative samples with a positive 16S result, these findings were supported by other diagnostic modalities, including blood cultures, additional biopsy specimens, or species-specific PCR assays (Table 3). For sample AD, no additional diagnostic modality could confirm presence of *Aggregatibacter* spp.; however, the clinical presentation of brain abscess was considered highly compatible and the 16S Nanopore-based sequencing result was considered a true positive. In sample AF, a tissue biopsy of a periprosthetic fracture, *Massilia timonae* was identified. Although this genus has been associated with postoperative infections, the clinical course was not suggestive of infection, as the patient improved without targeted antimicrobial therapy (Cho et al., 2017). While no corresponding reads were detected in negative controls, the possibility of environmental contamination cannot be excluded.

While the recovery of pathogens from culture-negative samples in this study was lower than reported in earlier studies (Lao et al., 2024; Vanhee et al., 2024), this study included a high proportion of tissue biopsies sampled due to suspicion of PJI. This could potentially affect recovery rates and the lack of objectivation of PJI diagnosis based on established criteria is a major limitation in the interpretation of these results (Olearo et al., 2025). An additional limitation is a possible deleterious effect of pre-analytical storage conditions (Range 0–14 days at 4 °C, mode 3 days) on the DNA stability and recovery of fastidious or low biomass organisms. While stability of total DNA extraction and proportional distribution of pathogens after short term storage at 4 °C have been described for higher biomass samples such as gingival plaque and feces, this is a potential source of bias and may underestimate sensitivity (Katsoulis et al., 2005).

As described, the interpretation of 16S rRNA gene sequencing results requires careful integration with clinical and conventional microbiological findings. Sequencing data alone do not distinguish between infection, colonization or contamination. In our setting, multidisciplinary discussion involving molecular biologists and medical microbiologists is used to contextualize sequencing findings appropriately. Interpretation is based on read abundance in correlation to the spike-in control (e.g., at least 20% of *Allobacillus halotolerans* reads) alongside a review of the clinical context, culture results and other diagnostic assays. Without careful consideration, there is a risk of both overdiagnosis and underdiagnosis of infection, which may lead to inappropriate antimicrobial use or delayed treatment. Accordingly, we believe 16S sequencing should be regarded as an adjunct to, rather than a replacement for, established diagnostic pathways (Olearo et al., 2025).

The 16S sequencing workflow presented here is cost-effective at €88 per sample when batching eight samples (including reagent-, flow cell-, data-analysis- and labor cost), mainly thanks to the use of the flongle flow cell. Nevertheless, the cost can vary from €162/sample when only a single sample is run (i.e., urgent samples) to €83 per sample when 16 samples are batched in a single run (Supplementary Table 4). In addition, the workflow presented here takes <7 h from DNA extraction to initiation of sequencing, even allowing same-day results to be reported when performing intermediate data-analysis in case of urgent samples. In addition, the use of the microbial amplicon barcoding kit allows ITS sequencing to be performed with the same protocol and even within the same run, although the latter is not recommended by the manufacturer due to inequivalent molarity when combining 16S and ITS amplicons without normalization.

This study demonstrates that the implementation of 16S rRNA sequencing is feasible in a routine clinical laboratory setting and provides added value for diagnostic microbiology, especially in culture negative and polymicrobial infections. While the workflow showed robust performance, cautious interpretation of sequencing results is warranted to avoid overinterpretation and misdiagnosis. Overall, these findings support 16S sequencing as a valuable adjunct to standard diagnostics, with clear potential for further optimization and broader clinical integration.

## Data Availability

All data not included in the paper, including raw read data for clinically relevant species, are available upon reasonable request. Raw sequencing data for the documented strains and clinical samples were submitted to the Sequence Read Archive (SRA) under the BioProject accession numbers PRJNA1479537 and PRJNA1479674, respectively.
