# Supplementary material for: Validation of a long-read 16S rRNA-gene sequencing approach for analysis of clinical samples and bacterial identification in a routine clinical laboratory
Source: Front Microbiol. 2026 Jul 30;17:1870167. doi: 10.3389/fmicb.2026.1870167 (PMC13496011; doi:10.3389/fmicb.2026.1870167)
Supplement: Supplementary file 1 [file Data_Sheet_1.docx]

**Supplementary Material**

**Validation of a long-read 16S rRNA-gene sequencing approach for analysis of clinical samples and bacterial identification in a routine clinical laboratory**

Van de gaer Otto^1^, Cartuyvels Reinoud1, Froyen Timo^1^, Hilkens Petra^1^, Magerman Koen^1,2^, Martens Steven^1^, Van Meensel Britt^1^, Dingemans Jozef^1^

**Figure S1**. Standard curves of the log_10_ read numbers in function of the theoretical load (using a 10-fold dilution series of a quantified bacterial suspension) for *S. aureus* in CSF (**A**), *P. aeruginosa* in CSF (**B**), *S. aureus* in synovial fluid (**C**) and *P. aeruginosa* in synovial fluid (**D**). The total number of reads (per sample) was normalized to 2500 to allow standardization between experiments. The estimated LoD at a cut-off of 1% (25 reads) of the total number of bacterial reads (2500 reads) is indicated via the red-dotted lines.

**Table S1.** Overview of the read numbers for *S. aureus* in CSF, *P. aeruginosa* in CSF, *S. aureus* in synovial fluid, and *P. aeruginosa* in synovial fluid when the total amount of reads was normalized to 2500 and the corresponding estimated LoD (based on the standard curves in Figure S1).

**Table S2.** Theoretical vs observed composition for the ZymoBIOMICS Microbial Community Standard (Zymo) per species.

| Species | Theoretical composition  based on 16S | Observed composition |
| --- | --- | --- |
| *Pseudomonas aeruginosa* | 4,20% | 2,70% |
| *Escherichia coli* | 10,10% | 13,40% |
| *Salmonella enterica* | 10,40% | 14,70% |
| *Lactobacillus fermentum* | 18,40% | 12,60% |
| *Enterococcus faecalis* | 9,90% | 9,10% |
| *Staphylococcus aureus* | 15,50% | 13,40% |
| *Listeria monocytogenes* | 14,10% | 10,20% |
| *Bacillus subtilis* | 17,40% | 13,20% |

**Table S3.** Overview of the intra-run and inter-run variation in relative abundance for the 5 most abundant species in the ZymoBIOMICS Microbial Community Standard II (Log Distribution).

| **Species** | **Relative abundance^a^**  **(run 1)** | **Relative abundance^a^**  **(run 2_A)** | **Relative abundance^a^**  **(run 2_B)** | **CV** |
| --- | --- | --- | --- | --- |
| ***Listeria monocytogenes*** | 82.0% | 86.4% | 86.8% | 3.1% |
| ***Pseudomonas aeruginosa*** | 15.0% | 11.1% | 10.6% | 19.7% |
| ***Bacillus subtilis*** | 1.1% | 0.8% | 1.0% | 15.8% |
| ***Escherichia coli*** | 0.3% | 0.2% | 0.3% | 21.7% |
| ***Salmonella enterica*** | 0.3% | 0.3% | 0.2% | 21.7% |

CV, variation coefficient.

**Table S4.** Overview of the effect of batch-size on the calculated cost of 16S rRNA sequencing using the workflow described in this study.

| Total cost on a Flongle flow cell | | | | | |
| --- | --- | --- | --- | --- | --- |
| # samples/run | **16** | **8** | **4** | **2** | **1** |
| Price (incl. VAT) | **€ 83** | **€ 88** | **€ 99** | **€ 120** | **€ 162** |
